# Self-supervised attention-based deep learning for pan-cancer mutation prediction from histopathology

**DOI:** 10.1038/s41698-023-00365-0

**Published:** 2023-03-28

**Authors:** Oliver Lester Saldanha, Chiara M. L. Loeffler, Jan Moritz Niehues, Marko van Treeck, Tobias P. Seraphin, Katherine Jane Hewitt, Didem Cifci, Gregory Patrick Veldhuizen, Siddhi Ramesh, Alexander T. Pearson, Jakob Nikolas Kather

**Affiliations:** 1 Else Kroener Fresenius Center for Digital Health, Medical Faculty Carl Gustav Carus, Technical University Dresden, Dresden, Germany; 2 Department of Medicine III, University Hospital RWTH Aachen, Aachen, Germany; 3 Department of Gastroenterology, Hepatology and Infectious Diseases, University Hospital Duesseldorf, Medical Faculty at Heinrich-Heine-University Duesseldorf, Düsseldorf, Germany; 4 Pritzker School of Medicine, University of Chicago, Chicago, IL USA; 5 Biological Sciences Division, University of Chicago, Chicago, IL USA; 6 University of Chicago Comprehensive Cancer Center, University of Chicago, Chicago, IL USA; 7 Pathology & Data Analytics, Leeds Institute of Medical Research at St James’s, University of Leeds, Leeds, UK; 8 Medical Oncology, National Center for Tumor Diseases (NCT), University Hospital Heidelberg, Heidelberg, Germany; 9 Department of Medicine 1, University Hospital and Faculty of Medicine Carl Gustav Carus, Technische Universität Dresden, Dresden, Germany

**Keywords:** Computational biology and bioinformatics, Diagnostic markers, Mathematics and computing

## Abstract

The histopathological phenotype of tumors reflects the underlying genetic makeup. Deep learning can predict genetic alterations from pathology slides, but it is unclear how well these predictions generalize to external datasets. We performed a systematic study on Deep-Learning-based prediction of genetic alterations from histology, using two large datasets of multiple tumor types. We show that an analysis pipeline that integrates self-supervised feature extraction and attention-based multiple instance learning achieves a robust predictability and generalizability.

The genotype of any solid tumor determines its phenotype, giving rise to a large variety of patterns in cancer histopathology. Deep learning (DL), a tool from the realm of artificial intelligence, can infer genetic alterations directly from routine histopathology slides stained with hematoxylin and eosin (H&E)^1,2^. Initial studies demonstrated this predictability in lung cancer^3^, breast cancer^4^, and colorectal cancer^5^. Subsequently, several “pan-cancer” studies showed that DL-based prediction of biomarkers is feasible across the whole spectrum of human cancer^6–10^. However, these studies were overwhelmingly performed in a single large cohort without externally validating the results on a large scale. This raises a number of potential concerns, as prediction performance can be heavily biased by batch effects occurring in such single multicentric datasets^11,12^. To move closer to clinical applicability, external validation of any DL system is paramount^13^. Recent technical benchmark studies have demonstrated that attention-based multiple instance learning (attMIL)^14^ and self-supervised learning (SSL)^15,16^ for pre-training of feature extractors^17,18^ can improve performance and generalizability for computational pathology biomarkers, but these technical advances have not yet been systematically applied to mutation prediction in a pan-cancer approach.

We acquired two large, multi-centric datasets of cancer histopathology images with matched genetic profiling: the Cancer Genome Atlas (TCGA) and the Clinical Proteomic Tumor Analysis Consortium (CPTAC) dataset. We used all tumor types which were present in both datasets, namely: breast (BRCA; TCGA *N* = 1066, CPTAC *N* = 122), colorectal (CRC; TCGA *N* = 534, CPTAC *N* = 105), glioblastoma (GBM; TCGA *N* = 397, CPTAC *N* = 96), lung adeno (LUAD; TCGA *N* = 566, CPTAC *N* = 110, lung squamous (LUSC; TCGA *N* = 484, CPTAC *N* = 108), pancreatic (PAAD; TCGA *N* = 179, CPTAC *N* = 140 patients), and (uterine) endometrial cancer (UCEC; TCGA *N* = 517, CPTAC *N* = 95; Fig. 1A, B and Supplementary Fig. 1A, B). We aimed to use Deep Learning to predict all *N* = 1068 clinically relevant oncogenes and tumor suppressor genes (Fig. 1C and Supplementary Fig. 1C) in the OnkoKb^19^ database. The number of genes analyzed decreased after excluding alterations of unknown significance and the definition of a minimum number of mutated cases. We trained the model to predict mutations in TCGA (Fig. 1A) and evaluated the performance on CPTAC (Fig. 1B). The primary endpoint was the mean (±standard deviation) area under the receiver operating characteristic curve (AUROC) of five replicate experiments. We benchmarked our methods against four other methods, as laid down in the “Methods”" section, and found that the combination of SSL + attMIL outperforms other tested approaches.

**Fig. 1:**
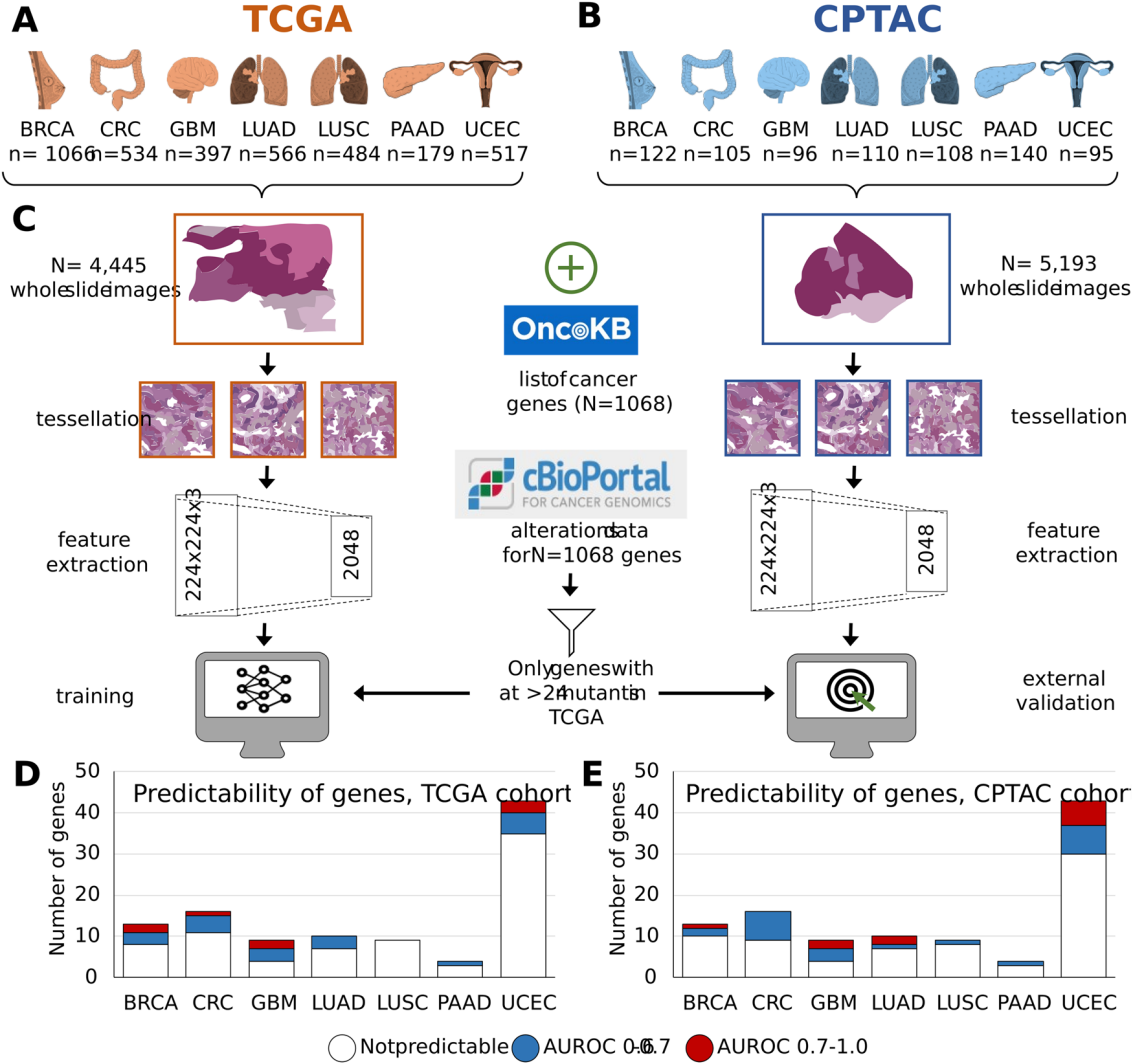
Study Design with selection process of the genes. **A**, **B** Patient numbers for each tumor type in The Cancer Genome Atlas (TCGA) and the Clinical Proteomic Tumor Analysis Consortium (CPTAC) dataset. **C** Flowchart showing the preprocessing steps for the training and validation cohort. Furthermore, an outline of the gene selection process. **D** Overview Area under the receiving operating curve (AUROC) results for internal cross-validation in TCGA. **E** Overview AUROC results for external validation on CPTAC. The plots are based on the original AUROC values with 5 decimal digits, while numbers in the manuscript text have been rounded to two decimal digits. (Icons were used from Servier Medical Art provided by Servier, licensed under a Creative Commons Attribution 3.0 unported license).

We found that in most tumor types, mutations in several genes were predictable from histology (Fig. 1D, E and Supplementary Data 1). In accordance with previous studies^20^, endometrial cancer (UCEC) had the highest number of detectable mutations. *N* = 13 out of n = 43 analyzable genes had an AUROC of 0.60 or higher, of which 6 reached an AUROC of 0.70 or higher in the external validation cohort (Fig. 2A). Among these were *PTEN* mutations (AUROC 0.73 ± 0.03) involved in hereditary cancer;^21^ *TP53* mutations (AUROC 0.72 ± 0.05), which is associated with poor prognosis^22^ and *APC* (AUROC 0.72 ± 0.11), as a potential predictive marker for immunotherapy in endometrial cancer^23^. We identified 7 genes for which mutations were predictable (out of 16 analyzable genes) for colorectal cancer (CRC) with AUROCs of over 0.6 in the external validation cohort. (Fig. 2B). This included prognostic alterations, such as *BRAF* and *KRAS* mutations, which reached an AUROC of 0.66 ± 0.24 and 0.66 ± 0.03 respectively (Supplementary Data 1). In GBM, 4 out of 9 genes had an AUROC over 0.70 including the genes *IDH1* (AUROC 0.84 ± 0.06), *ATRX* (AUROC 0.70 ± 0.10), *TP53* (AUROC 0.70 ± 0.07) and *RB1* (AUROC 0.70 ± 0.07; Fig. 2C). These are of increasing relevance as classification of brain tumors are increasingly based on molecular markers and therefore of therapeutic importance. In the other tumor types prognosis relevant mutations such as *EGFR* in LUAD (AUROC 0.76 ± 0.03) or *TP53* in BRCA (AUROC 0.72 ± 0.05) could be predicted with an AUROC over 0.70 (Fig. 2D, E). For the tumor type LUSC highest AUROCS of 0.61 ± 0.14 for *RB1* was achieved (Fig. 2F). Moreover the DL algorithm was able to detect CDH1 alterations in BRCA with an AUROC of 0.68 ± 0.17 (Fig. 2G–I). Compared to the other tumor types in our study, the tumors with the highest number of predictable alterations (UCEC, CRC, and BRCA) have a higher average tumor mutational burden^24^. We hypothesize that many morphological alterations are related to immune-mediated changes in the tumor microenvironment. Our method yielded interpretable spatial predictions (Fig. 2G), and unlike previous studies^25^ provided separate heatmaps for attention (Fig. 2H) and classification (Fig. 2I and Supplementary Fig. 2). Previous studies have shown such heatmaps to be correlated to the underlying molecular ground truth on a spatial scale^26,27^. In summary, in our study, the use of the new combination of SSL + attMIL showed the best performance in comparison to the other techniques (Supplementary Fig. 3 and Supplementary Data 2), while a visual examination of correctly and wrongly classified cases suggested a plausible distribution of model attention on the whole slide image (Supplementary Figs. 4A–O, 5A–O, 6A–O, and 7A–O).

**Fig. 2:**
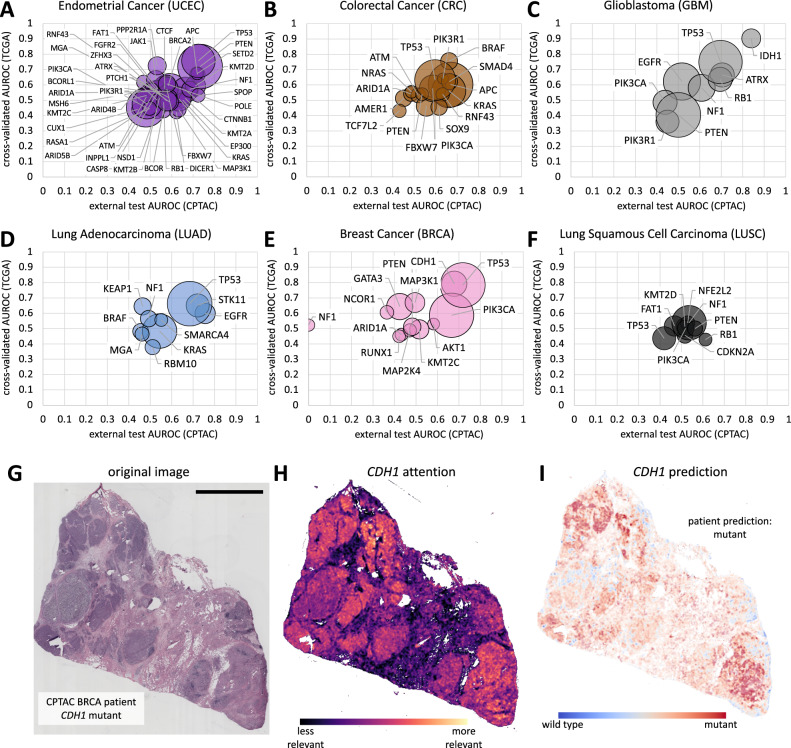
Classification performance for all genes in internal and external validation. **A**–**F** Internal cross-validation and external validation Area under the receiving operating curve (AUROC) for six tumor types (PAAD in Supplementary Fig. 8). The bubble size scales with the number of mutant patients in the external validation cohort. All raw data are in Supplementary Data 1. **G**–**I** A representative patient from the CPTAC-BRCA cohort, with attention map and prediction maps for *CDH1* mutational status. The scale bar represents 5 mm.

A key limitation is that many clinically relevant genes were not analyzable due to having fewer than 25 mutants in TCGA or many alterations with unknown significance. Large-scale efforts are needed to create datasets with a sufficient size, which could be facilitated by federated^28^ or swarm^29^ learning. Since the early 2000s, studies have shown a link between genetic alterations and histological phenotypes^30^, which DL can exploit^1,6,7^. While there is no biological reason why every frequent genetic alteration is actually manifest in histology, our results add to the growing amount of evidence which shows that many of these alterations are indeed determinable from H&E. This is also detached from the morphological subtype (Supplementary Fig. 3B) or other molecular alterations such as MSI or *POLE* mutational status (Supplementary Fig. 3C–E). Crucially, in contrast to previous studies, our pan-cancer mutation prediction models have been externally validated, thereby minimizing the risk of overfitting^11^.

Our analysis shows that in almost all tumor types, cross-validated performance of SSL + attMIL is correlated to external validation performance (Fig. 2A–F and Supplementary Fig. 8) and outperforms the current state of the art. To our knowledge, this is the first time that a multiplexed biomarker prediction from H&E slides has been shown to generalize well. Our study identifies a number of clinically relevant candidate genes amenable to DL-based pre-screening as part of clinical routine practice, with the aim of identifying patients who are good candidates for confirmatory genetic testing.

## Methods

### Ethics statement

All experiments were conducted in accordance with the Declaration of Helsinki. For this study, we used anonymized H&E-stained slides from public repositories.

### Data acquisition and experimental design

Mutation data for was obtained from https://www.cbioportal.org/^31^, excluding alterations of unknown significance and excluding all genes with fewer than *N* = 25 mutant cases in TCGA (Fig. 1C and Supplementary Fig. 1C). This resulted in 43 analyzable genes in endometrial cancer (UCEC), down to 4 analyzable genes in pancreatic cancer (PAAD, Fig. 2A–F and Supplementary Fig. 8). We then used our in-house open-source DL pipeline (https://github.com/KatherLab/marugoto) which uses the SSL-trained model RetCCL^32^ to obtain 2048 features per tile and uses attMIL to make patient-level predictions^33,34^.

### Model architecture

The methods used in this paper follow a two-step approach: the first step is the feature extraction (transforming image tiles into feature vectors) and the second step is slide aggregation (transforming a set of feature vectors obtained from a given pathology slide into a single prediction for that slide).

For feature extraction, we explore two different methods using a Resnet neural network which was pretrained in a different way. The first feature extraction model is based on a Resnet18 which was pre-trained on ImageNet^35^. We chose this model due to its broad use in the computational pathology research literature^14^. The second model is the Retrieval with Clustering-guided Contrastive Learning (RetCCL)^32^ model, a Resnet50 backbone that was trained on a pathology dataset with Self Supervised Learning (SSL).

Also, for the aggregation, we explore two different methods: average pooling (avgPool), which was the dominant approach in the 2018–2020 research literature on clinical datasets^3,5,14,36^. This approach uses a simple multilayer perceptron similar to obtain a prediction for each tile, and then averages the predictions across all tiles for a given slide. The architecture of the multilayer perceptron (classifier network) is (512 × 256), (256 × 2). In contrast, the attention-based MIL model (attMIL^33^) has the following architecture: (512 × 256), (256 × 2) with an additional attention mechanism^37^. Finally, we use hyperbolic tangent (tanh) as an activation layer to obtain a prediction score.

The core method of our in-house image analysis pipeline “marugoto” is to combine an SSL feature extractor with an attMIL aggregation model. To benchmark both of the models against a baseline, we combine feature extraction and aggregation models in four different ways: ImageNet+attMIL, ImageNet+avgPool, SSL + attMIL, and SSL + avgPool. Lastly, we compared these new techniques to our previous in-house pipeline DeepMed^38^ which implements the approach proposed by Coudray et al.^3^. In this approach, the deep layers of a Resnet18 are fine-tuned on image tiles, as described before^5^. We apply all five approaches to the mutation prediction task in colorectal cancer (train on TCGA-CRC, test on CPTAC-CRC) and compare the results (Supplementary Fig. 3A). Based on these, we choose SSL + attMIL for all other experiments.

### Reporting summary

Further information on research design is available in the Nature Research Reporting Summary linked to this article.

## Supplementary information

Supplementary Material

Supplementary Data 1

Supplementary Data 2

REPORTING SUMMARY

## Data Availability

TCGA images are from https://portal.gdc.cancer.gov/, CPTAC images are from https://wiki.cancerimagingarchive.net/display/Public/CPTAC + Pathology+Slide+Downloads. Genetic data are available at https://www.cbioportal.org/.

## Code availability

Our pipeline is available under an open source license (https://github.com/KatherLab/marugoto).
