# Supplementary material for: Self-supervised attention-based deep learning for pan-cancer mutation prediction from histopathology
Source: NPJ Precis Oncol. 2023 Mar 28;7:35. doi: 10.1038/s41698-023-00365-0 (PMC10050159; doi:10.1038/s41698-023-00365-0)
Supplement: Supplementary file 1 — Supplementary Material [file 41698_2023_365_MOESM1_ESM.pdf]

Supplementary Figures

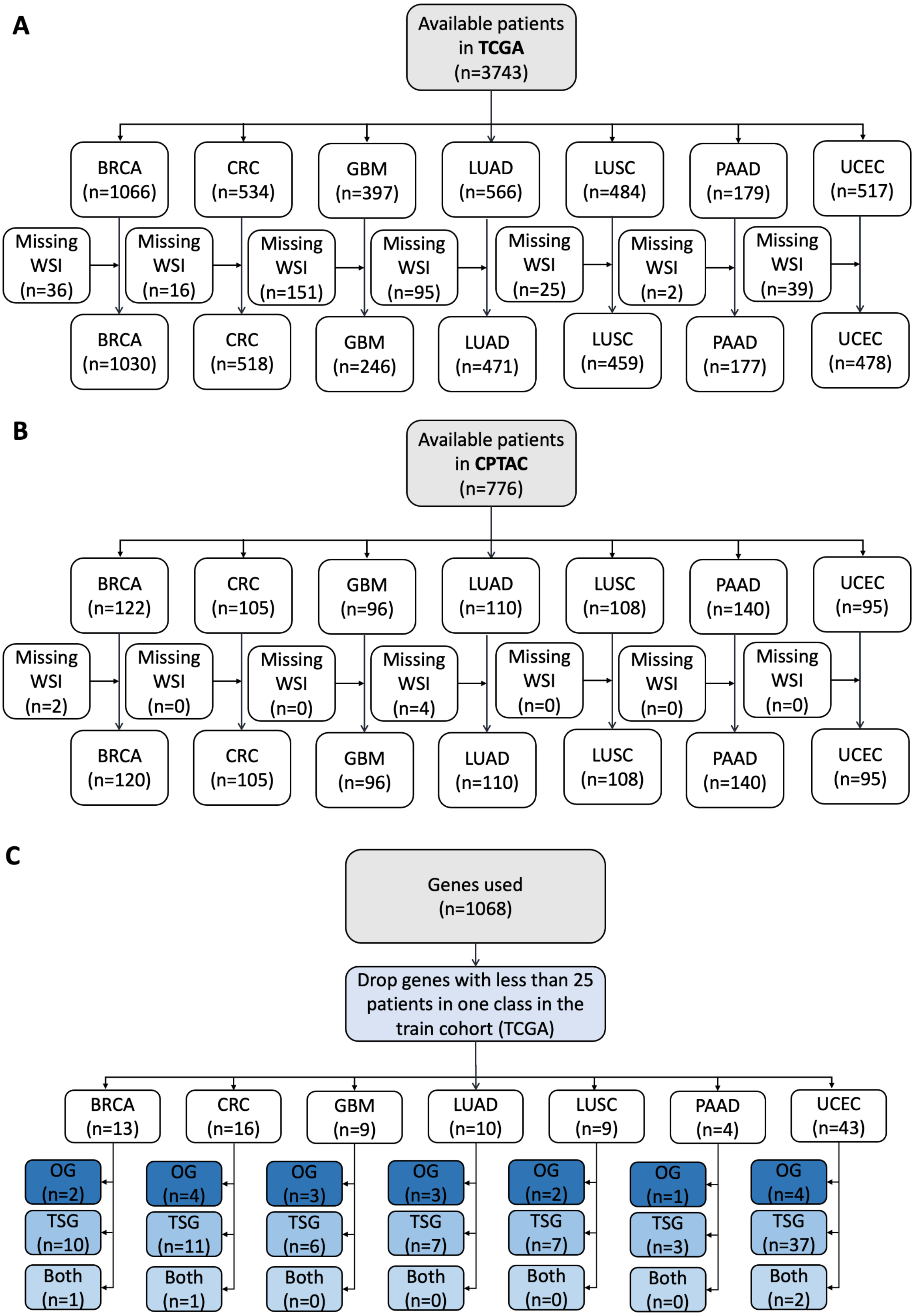

Supplementary Figure 1: CONSORT flowchart Diagrams. (A) Patient consort diagram for The Cancer Genome Atlas, (TCGA) cohort, **(B)** patient consort diagram for the Clinical Proteomic Tumor Analysis Consortium (CPTAC) cohort. “Missing status” means that no molecular information was available in the cbioportal database for this patient. **(C)** Selection process of genes included and count per tumor type and respective number of Oncogenes (OG), Tumor suppressor gene (TSG) or both. BRCA = breast invasive carcinoma; CRC = colorectal carcinoma; GBM = glioblastoma multiforme; LUAD = lung adenocarcinoma; LUSC = lung squamous cell carcinoma; PAAD = pancreatic adenocarcinoma; UCEC = uterine corpus endometrial carcinoma

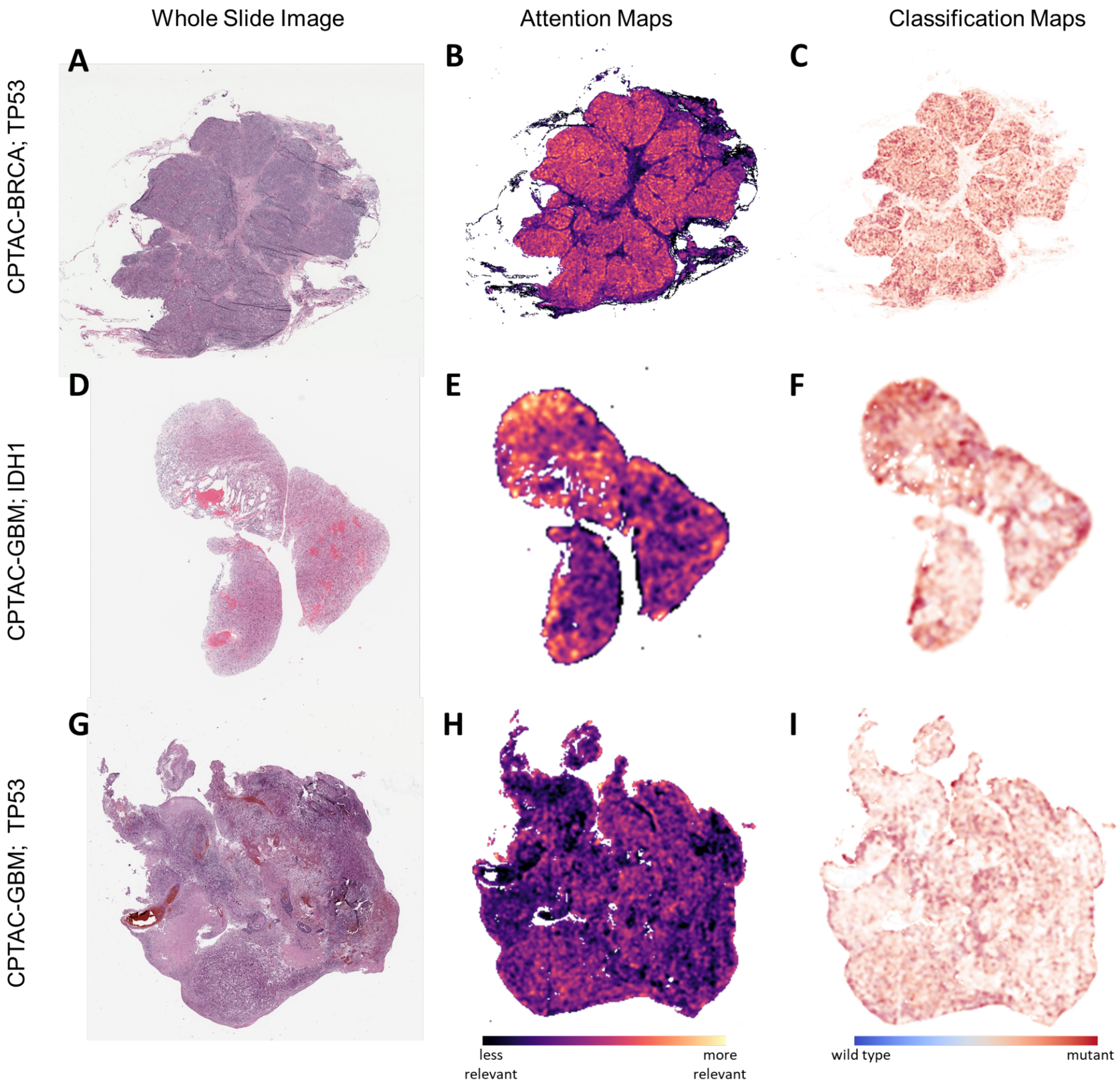

Supplementary Figure 2: Representative attention and prediction maps. The procedure for visualization and the color maps are identical to **Figure 2G-I**, respectively. Whole-slide Images, attention and classification Maps (ground truth: mutated, prediction: mutated) are shown for **(A-C)** *TP53* Mutation in CPTAC-BRCA; **(D-F)** *IDH1* mutation in CPTAC-GBM; **(G-I)** *TP53* mutation in CPTAC-GBM. This slide has been cropped for visualization purposes (the original slide had two near-identical pieces of tissue on it).

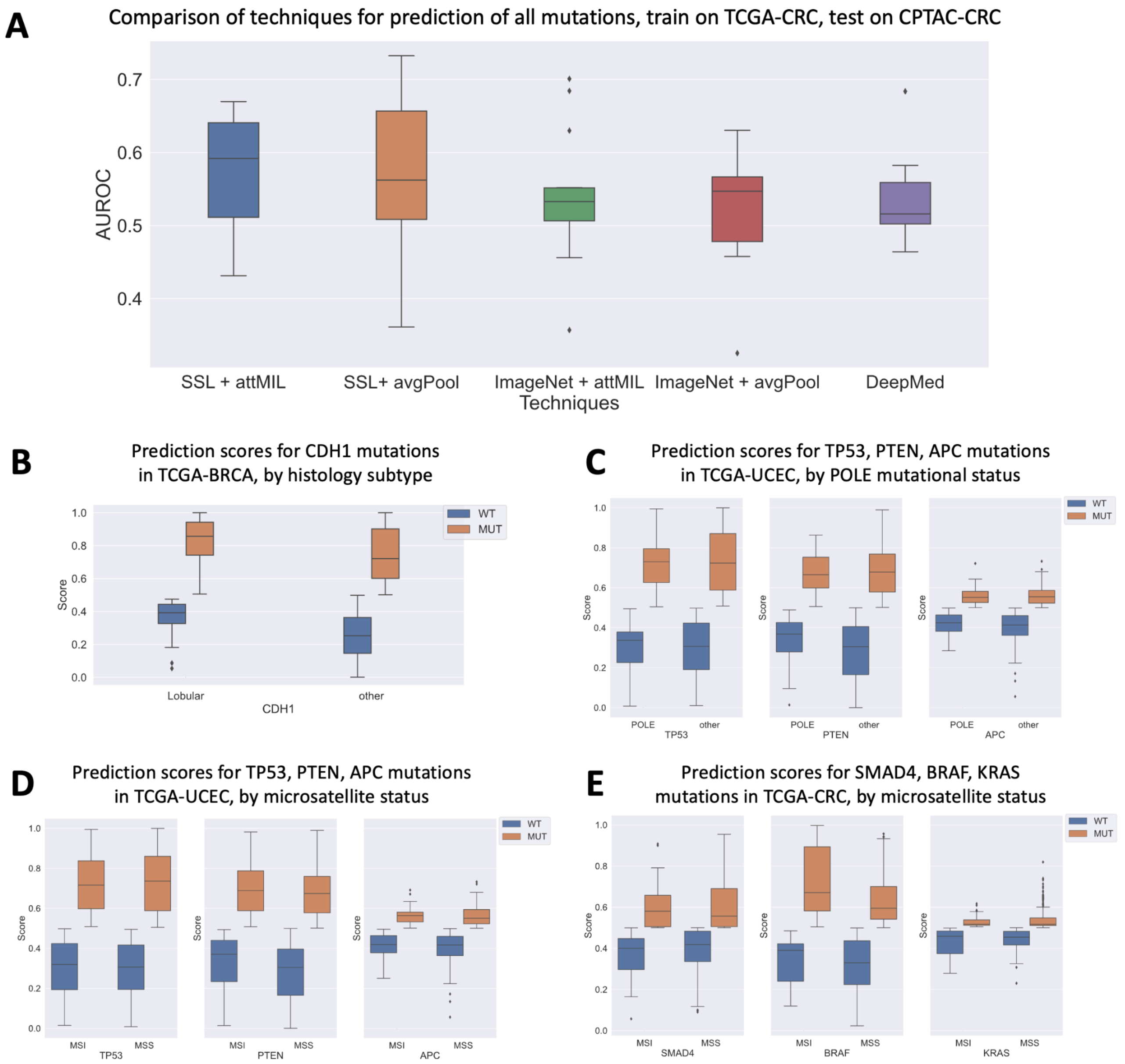

Supplementary Figure 3: Box Plot of Area under the receiving operating curve: (A) AUROC for patient level prediction for the 16 genes validated in the Clinical Proteomic Tumor Analysis Consortium CPTAC- CRC cohort. The Box plot compares the performance of different techniques: self-supervised learning (SSL) + attention Multiple Instance Learning (attMIL), SSL + average pooling (avgPool), ImageNet+attMIL, ImageNet+avgPool and DeepMed. **(B)** Area under the receiving operating curve (AUROC) for patient level prediction of *CDH1* mutational status in The Cancer Genome Atlas (TCGA) - breast carcinoma (BRCA) lobular and other subtypes. **(C-D)** AUROC for patient level prediction of *TP53, PTEN* and *APC* mutational status in TCGA-UCEC (endometrial cancer) with **(C)** *POLE* alteration or other; **(D)** microsatellite instable (MSI) and microsatellite stable (MSS). **(E)** AUROC for patient level prediction of *SMAF4, BRAF* and *KRAS* mutational status in TCGA-CRC colorectal for MSI and MSS. The box represents the interquartile range (IQR) between the first and third quartile and the horizontal line inside the box indicates the median. The whiskers extend from the minimum and maximum data point within 1.5 times the IQR. Outlier data points are represented by individual markers.

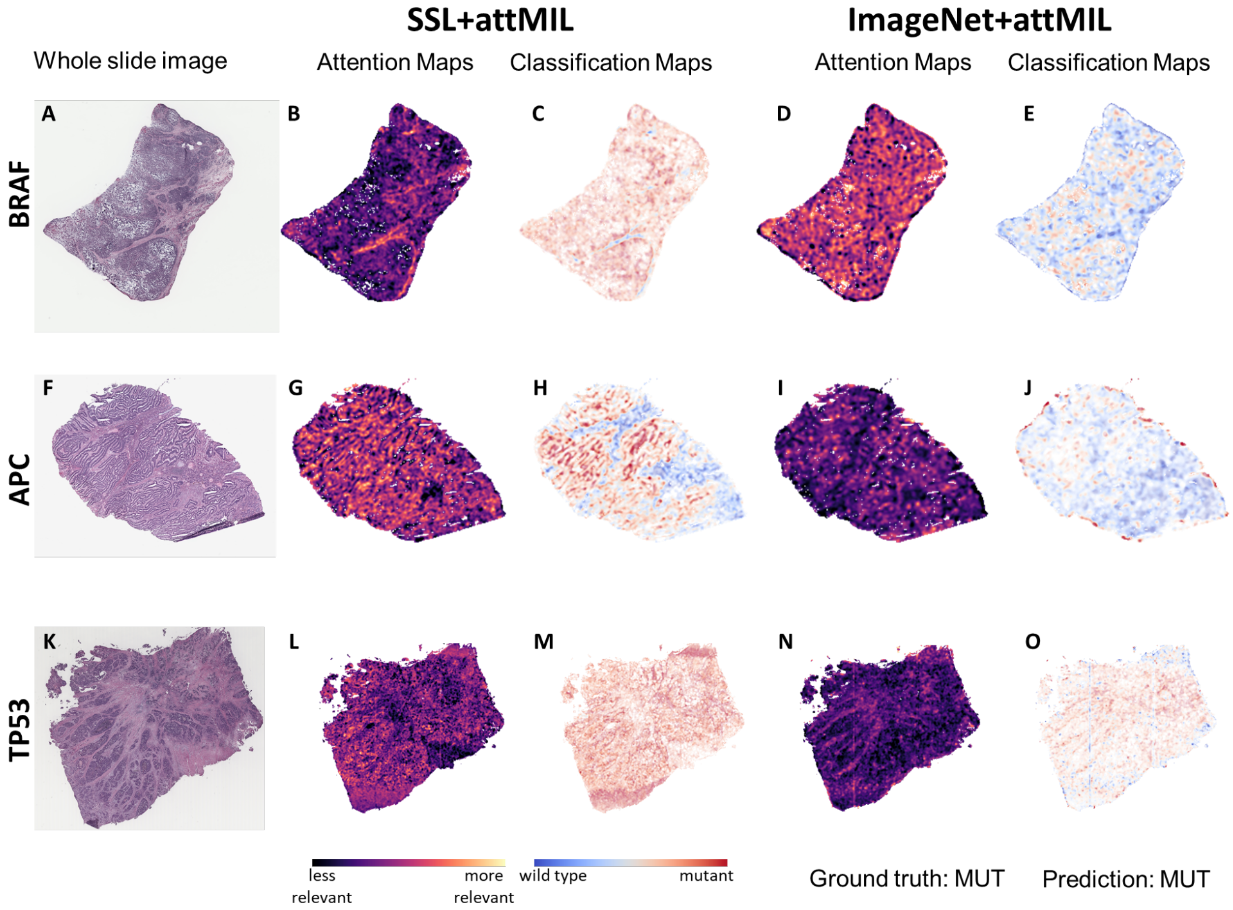

Supplementary Figure 4: Attention and Classification maps for different techniques #1: Whole-slide Images, attention and classification maps (ground truth: mutated (MUT), prediction: MUT) comparing the self-supervised learning (SSL) + attention Multiple Instance Learning (MIL) method to the ImageNet + attMIL approach are shown for **(A-E)** *BRAF* Mutation in Clinical Proteomic Tumor Analysis Consortium (CPTAC) - CRC; **(F-J)** *APC* Mutation in CPTAC - CRC; **(K-O)** *TP53* Mutation in CPTAC - CRC. These slides have been cropped for visualization purposes (the original slide had two near-identical pieces of tissue on it).

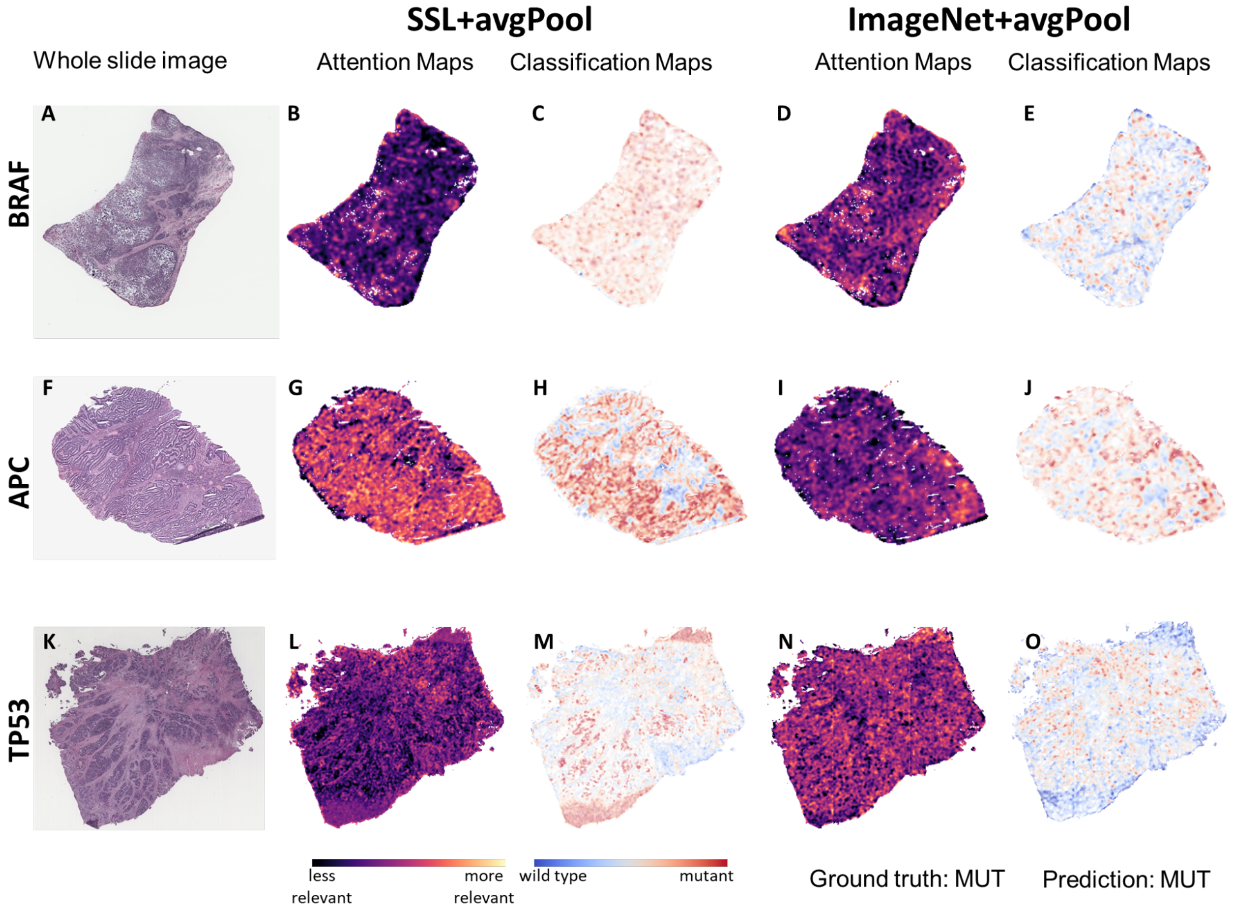

Supplementary Figure 5: Attention and Classification maps for different techniques #2: Whole-slide Images, attention and classification maps (ground truth: mutated (MUT), prediction: MUT comparing the self-supervised learning (SSL) + average pooling (avgPool) method to the ImageNet + avgPool approach are shown for **(A-E)** *BRAF* Mutation in Clinical Proteomic Tumor Analysis Consortium (CPTAC) - CRC; **(F-J)** *APC* Mutation in CPTAC - CRC; **(K-O)** *TP53* Mutation in CPTAC - CRC. These slides have been cropped for visualization purposes (the original slide had two near-identical pieces of tissue on it).

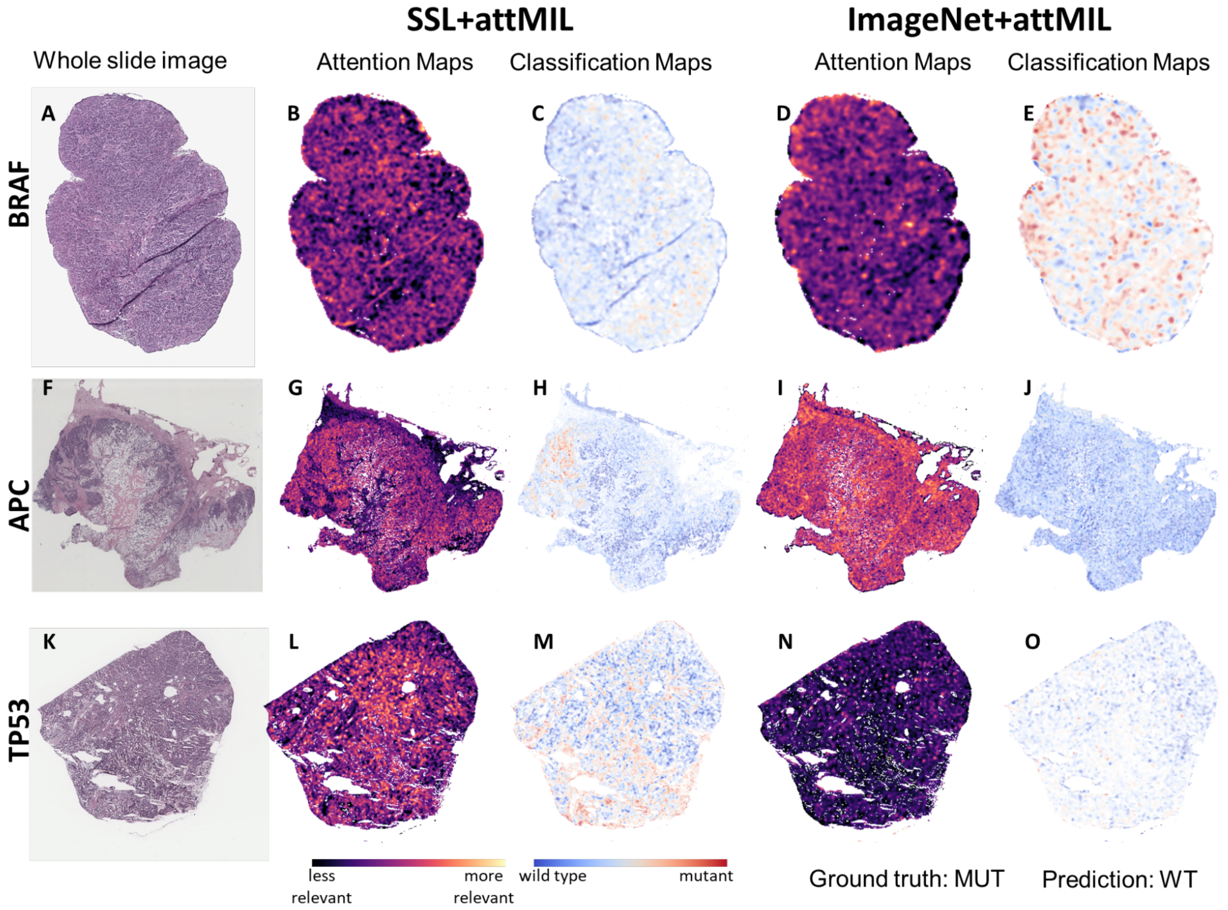

Supplementary Figure 6: Attention and Classification maps for different techniques #3: Whole-slide Images, attention and classification maps (ground truth: mutated (MUT), prediction: wildtype (WT)) comparing the self-supervised learning (SSL) + attention Multiple Instance Learning (MIL) approach to the ImageNet + attMIL approach are shown for **(A-E)** *BRAF* Mutation in Clinical Proteomic Tumor Analysis Consortium (CPTAC) - CRC; **(F-J)** *APC* Mutation in CPTAC - CRC; **(K-O)** *TP53* Mutation in CPTAC - CRC. These slides have been cropped for visualization purposes (the original slide had two near-identical pieces of tissue on it).

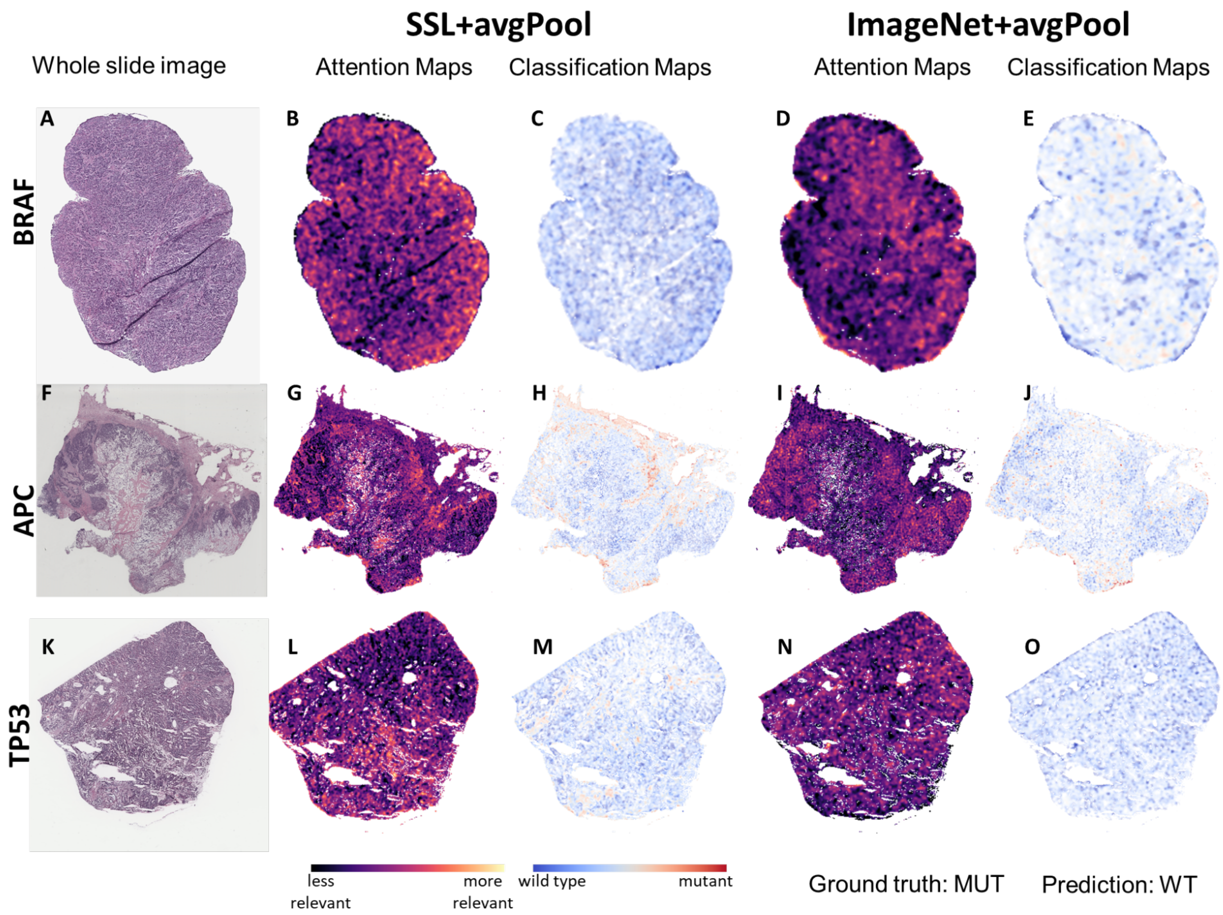

Supplementary Figure 7: Attention and Classification maps for different techniques #4: Whole-slide Images, attention and classification maps (ground truth: mutated, prediction: mutated) comparing the self-supervised learning (SSL) + average pooling (avgPool) method to the ImageNet + avgPool approach are shown for **(A-E)** *BRAF* Mutation in Clinical Proteomic Tumor Analysis Consortium (CPTAC) - CRC; **(F-J)** *APC* Mutation in CPTAC - CRC; **(K-O)** *TP53* Mutation in CPTAC - CRC. These slides have been cropped for visualization purposes (the original slide had two near-identical pieces of tissue on it).

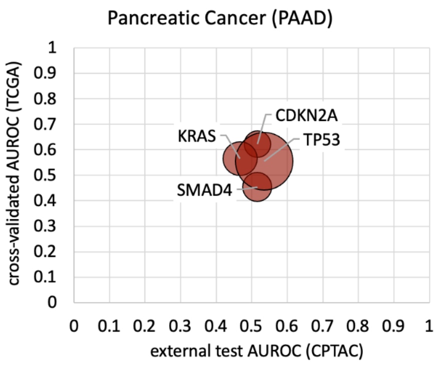

Supplementary Figure 8: Prediction results for pancreatic cancer (PAAD). In PAAD, only 4 genes were analyzable because only these genes met the inclusion criterion of having at least 25 mutant cases in the training cohort. All genes were predictable with a poor Area under the receiving operating curve (AUROC) of <0.6 in the internal and external cohort.
